# Discerning Neurogenic vs. Non-Neurogenic Postnatal Lateral Ventricular Astrocytes via Activity-Dependent Input

**DOI:** 10.3389/fnins.2016.00111

**Published:** 2016-03-24

**Authors:** Elena W. Adlaf, Aaron Mitchell-Dick, Chay T. Kuo

**Affiliations:** ^1^Department of Cell Biology, Duke University School of MedicineDurham, NC, USA; ^2^Cellular and Molecular Biology Graduate Training Program, Duke University School of MedicineDurham, NC, USA; ^3^Brumley Neonatal Perinatal Research Institute, Duke University School of MedicineDurham, NC, USA; ^4^Department of Neurobiology, Duke University School of MedicineDurham, NC, USA; ^5^Preston Robert Tisch Brain Tumor Center, Duke University School of MedicineDurham, NC, USA; ^6^Duke Institute for Brain Sciences, Duke UniversityDurham, NC, USA

**Keywords:** adult neurogenesis, neural stem cells (NSCs), cholinergic circuit, lateral ventricles, astrogenesis

## Abstract

Throughout development, neural stem cells (NSCs) give rise to differentiated neurons, astrocytes, and oligodendrocytes which together modulate perception, memory, and behavior in the adult nervous system. To understand how NSCs contribute to postnatal/adult brain remodeling and repair after injury, the lateral ventricular (LV) neurogenic niche in the rodent postnatal brain serves as an excellent model system. It is a specialized area containing self-renewing GFAP^+^ astrocytes functioning as NSCs generating new neurons throughout life. In addition to this now well-studied regenerative process, the LV niche also generates differentiated astrocytes, playing an important role for glial scar formation after cortical injury. While LV NSCs can be clearly distinguished from their neuroblast and oligodendrocyte progeny via molecular markers, the astrocytic identity of NSCs has complicated their distinction from terminally-differentiated astrocytes in the niche. Our current models of postnatal/adult LV neurogenesis do not take into account local astrogenesis, or the possibility that cellular markers may be similar between non-dividing GFAP^+^ NSCs and their differentiated astrocyte daughters. Postnatal LV neurogenesis is regulated by NSC-intrinsic mechanisms interacting with extracellular/niche-driven cues. It is generally believed that these local effects are responsible for sustaining neurogenesis, though behavioral paradigms and disease states have suggested possibilities for neural circuit-level modulation. With recent experimental findings that neuronal stimulation can directly evoke responses in LV NSCs, it is possible that this exciting property will add a new dimension to identifying postnatal/adult NSCs. Here, we put forth a notion that neural circuit-level input can be a distinct characteristic defining postnatal/adult NSCs from non-neurogenic astroglia.

## Introduction

**Figure 1:**
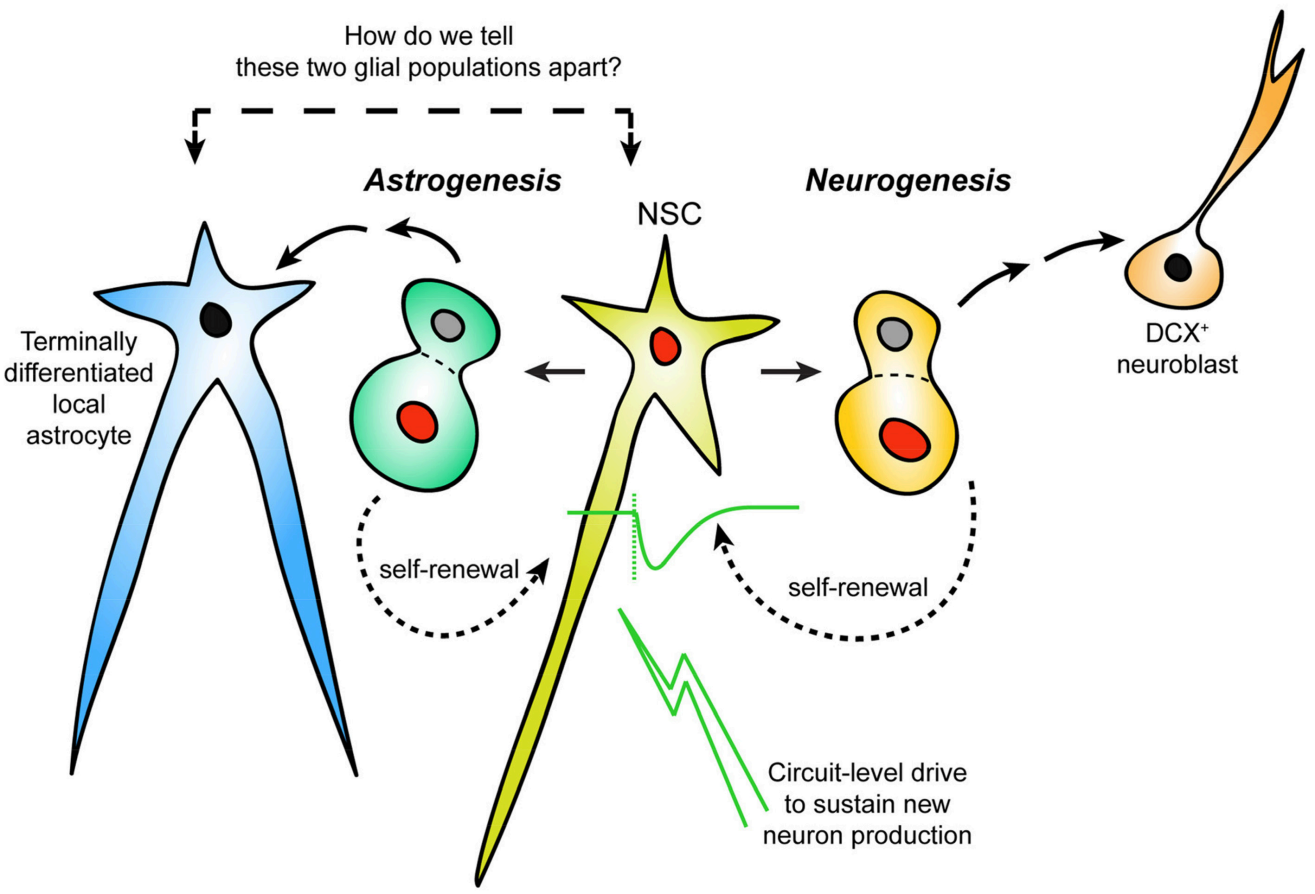
**Distinguishing neurogenic vs. non-neurogenic adult LV astrocytes**. Schematic representation of an area of postnatal/adult LV neurogenesis needing reconsideration: the incorporation of astrogenesis in the context of ongoing neurogenesis. It is currently unclear how newly-generated (but terminally-differentiated) local astrocytes can be distinguished from NSCs that are not actively dividing. Response to neuronal activation may separate LV NSCs from other niche astrocytes.

During embryonic neurogenesis, the brain is constructed in a systematic and reproducible way by the division of NSCs, and the migration/differentiation of their progeny (Ma et al., 2009; Urbán and Guillemot, 2014). The requirements for neurogenesis to persist in distinct regions of the adult mammalian brain, which include the subgranular zone (SGZ) of the hippocampus and the lateral wall of the LV, but not others, are still not fully understood. It is generally believed that proliferation of adult NSCs to generate new neurons serves the functional needs of established neural circuits in a region-specific and stimulus-dependent manner. Thus, it is possible that network activity, driven by environmental stimuli, instructs the proliferation, migration and differentiation of postnatal NSCs. In this fashion, postnatal/adult neurogenesis may actively contribute to neural plasticity via a stimuli-driven feedback loop, in contrast to embryonic neurogenesis, which operates on a well-tuned timer for reproducible anatomical construction. Classically, for a cell to be defined as an NSC, it should possess the ability to undergo asymmetrical cell division for both self-renewal and generation of new neurons. How to positively identify NSCs from a seemingly heterogeneous population of cell types in the postnatal/adult neurogenic niche presents a significant challenge for experimental design and data interpretation. Currently, the most utilized methods for identifying adult NSCs based on morphological and molecular methods are perhaps overly inclusive or exclusive depending on context. When we visualize a GFAP^+^ glia in the neurogenic niche, how do we tell whether it is neurogenic or not? What if the niche produced local, terminally-differentiated astrocytes with similar morphological and molecular characteristics as those defining NSCs? Our current models do not distinguish these important differences (Figure 1). This perspective summarizes emerging studies of LV astrogenesis as well as alternative strategies for defining postnatal NSCs and their potential drawbacks. We argue that circuit-level drive to sustain progenitor proliferation is an important aspect of adult neurogenesis/astrogenesis, and this property could be utilized to further define LV NSCs vs. terminally differentiated local astrocytes.

## Glial identity of LV NSCs

In a seminal 1999 study, Alvarez-Buylla and colleagues showed convincingly that a subset of LV cells expressing glial fibrillary acidic protein (GFAP) had the characteristics of NSCs (Doetsch et al., 1999a). GFAP^+^ cells in the LV niche (also termed type B cells) were labeled with proliferation markers over long survival periods, and an intraventricularly-injected retrovirus targeting GFAP^+^ cells resulted in labeled neuroblasts and neurons in the olfactory bulb. After elimination of proliferating LV cell types with the antimitotic agent Ara-C, GFAP^+^ cells remained in the niche, began to divide and could be traced as the precursors of Mash1^+^ transient amplifying cells (type C cells) and migrating neuroblasts (type A cells; Doetsch et al., 1999a; Alvarez-Buylla and Lim, 2004).

**Figure 2:**
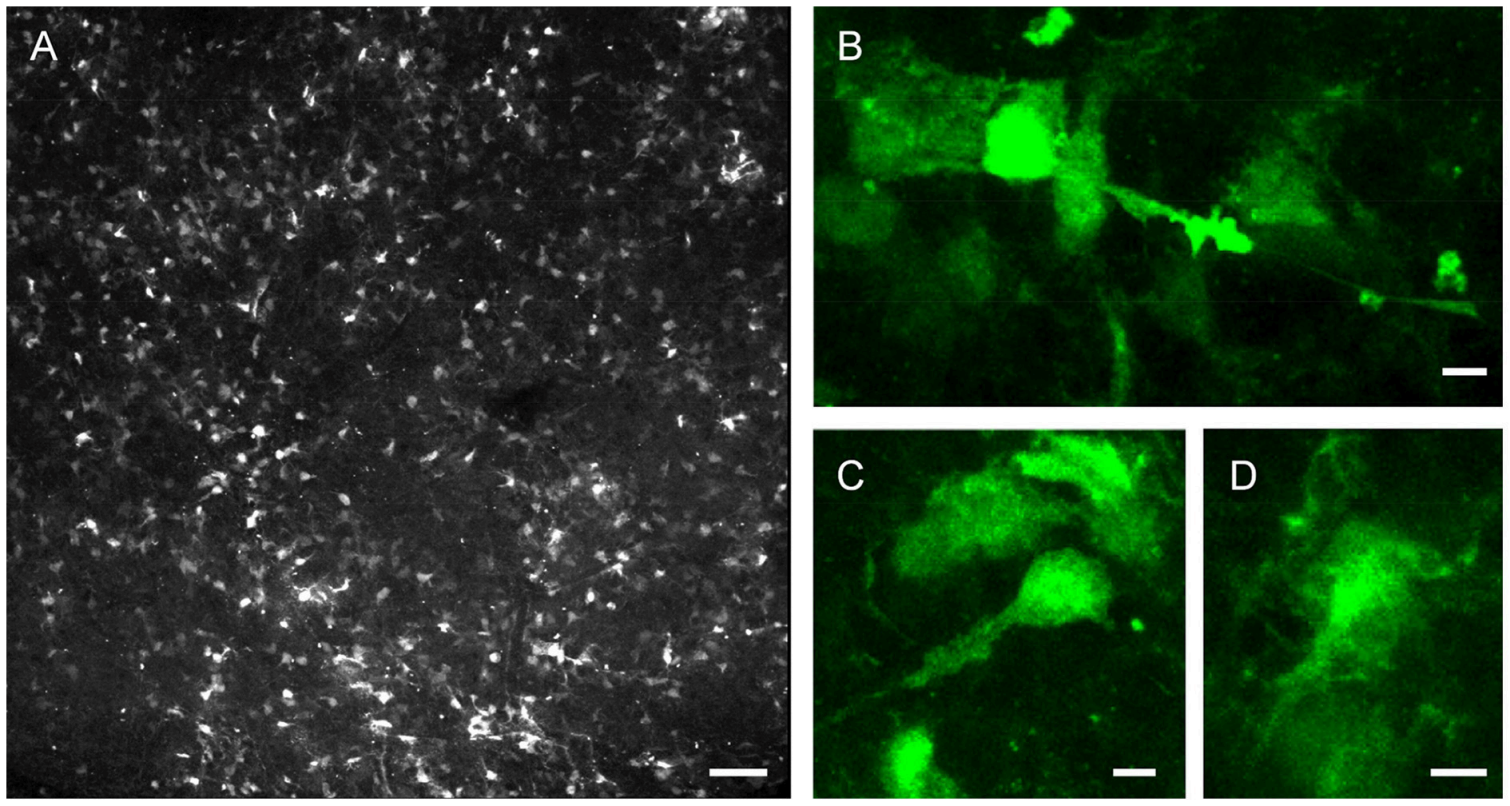
**Morphological diversity of postnatal LV niche GFAP-GFP^**+**^ cells**. LV lateral wall wholemount tissue preparation from P32 *GFAP-GFP* reporter mouse, imaged via endogenous GFP fluorescence. **(A)** Representative confocal enface view of lateral wall surface, at the level of anterior commissure, known to contain ependymal niche pinwheel-like structures. **(B–D)** Close-up views of example GFP^+^ subependymal cells. Note the differences in cellular morphologies. Bars = 100 μm **(A)**, 5 μm **(B–D)**.

In addition to the neurogenic subset of type B astrocytes, designated type B1, GFAP^+^ cells within the LV niche include type B2 astrocytes (García-Verdugo et al., 1998; Mirzadeh et al., 2008) and stellate astrocytes (Ma et al., 2005). These cell types are not always morphologically distinct (Garcia et al., 2004; Shen et al., 2008), and can be a challenge to distinguish during tissue experiments probing NSC function. In recent years, for simplicity, the process of adult LV neurogenesis has mostly been described in schematics to illustrate subependymal zone (SEZ) astrocytes functioning as NSCs. Figure 2 shows native GFP fluorescence (without antibody staining) from an LV wholemount harvested from adult *GFAP-GFP* animal, showing the difficulty in distinguishing different GFP^+^ cell types based on morphology in real time. For simplicity during experimentation using live cells, GFAP and other astrocytic markers, such as GLAST1, have nonetheless been generalized in many instances as positive identifiers of NSCs within the SEZ of the LV niche.

Progress in moving away from such generalized astrocytic markers has been hindered by a lack of reliable, alternative expression markers that can clearly distinguish neurogenic vs. non-neurogenic LV astrocytes (Mamber et al., 2013). Further complicating this problem, adult NSCs can become quiescent *in vivo* over long timespans and change their proliferative profile/markers in the process (Doetsch et al., 1999b; Codega et al., 2014; Calzolari et al., 2015). Single-cell sequencing technology can be a powerful tool for expression profiling of LV NSCs in different states. Combined with fluorescent activated cell sorting (FACS) using cell surface markers, these approaches may provide the necessary specificity to more accurately characterize NSCs (Pastrana et al., 2009; Mich et al., 2014; Llorens-Bobadilla et al., 2015). However, an important consideration is that multi-genetic fluorescence labeling are difficult/time-consuming to generate for use in live tissue experiments, such as *in vitro* recording or live cell imaging.

## Morphological definition of LV NSCs

Anatomical features of NSCs have been combined with GFAP expression to further refine the positional and morphological definition of a postnatal/adult LV NSC. B1 type astrocytes within the LV niche had originally been described to possess a primary cilium contacting the cerebrospinal fluid from the apical surface (Doetsch et al., 1997). Subsequent experiments revealed that they: (1) possess polarized and extended basal endfeet to contact blood vessels (Shen et al., 2008; Tavazoie et al., 2008); and (2) are arranged into a pinwheel-like architecture together with neighboring ependymal niche cells (Mirzadeh et al., 2008; Paez-Gonzalez et al., 2011). The combinatorial usage of astrocytic marker + anatomical features represents perhaps our current state-of-the-art in identifying LV niche NSCs in immunohistochemical experiments and their analyses. It has been well-described that the endfeet of stellate astrocytes also contact blood vessels and are an integral modulator of the blood-brain-barrier (Abbott et al., 2006). Also, the LV *medial* wall can be neurogenic (Merkle et al., 2007), although ependymal pinwheel-like niche structures have not been described in this brain region. Thus, it is difficult to conclude that contacting blood vessels and/or arranging into ependymal pinwheel structures are specific anatomical features for postnatal/adult LV NSCs.

## Genetic lineage-tracing of postnatal NSCs

While molecular markers and anatomical features are indispensable for NSC identification, they do not directly address the key cellular feature for these cells to generate neuronal progeny in the adult brain. Nestin is an intermediate filament protein expressed in nervous system cells during active division (Lendahl et al., 1990). To genetically define the cellular activity of postnatal/adult NSCs, we and others have generated tamoxifen-inducible Nestin-CreER transgenic drivers, together with Cre-driven reporters, to lineage-trace and understand the developmental process of neurogenesis (Kuo et al., 2006; Lagace et al., 2007; Aponso et al., 2008; Giachino and Taylor, 2009; Dhaliwal and Lagace, 2011; Benner et al., 2013; Faiz et al., 2015; Sohn et al., 2015). While this approach has been highly successful and widely adopted, Nestin-CreER also targets LV niche ependymal cells (Kuo et al., 2006), which are generally believed to be post-mitotic but express Nestin like their NSC counterpart. It is also important to note that, due to the nature of transgenic approaches, the different Nestin-CreER lines vary in NSC targeting efficiency as well as niche ependymal cells labeled (Kuo et al., 2006; Lagace et al., 2007; Giachino and Taylor, 2009). This labeling presents a significant challenge for NSC identification, as several publications have indicated neurogenic potential for ependymal niche cells under physiological and/or injury conditions (Johansson et al., 1999; Coskun et al., 2008; Carlén et al., 2009; Nomura et al., 2010; Luo et al., 2015).

GFAP-CreER and GLAST1-CreER lines have also been used to quantify the production of newborn neurons and oligodendrocytes from LV NSCs (Menn et al., 2006; Dhaliwal and Lagace, 2011; Calzolari et al., 2015). These drivers by definition will label mature astrocytes in the brain, and so they were used mainly to identify terminally-differentiated NSC progeny that had migrated away from the LV niche. However, these lines cannot clearly identify the cellular origins of newborn neurons or oligodendrocytes within the LV niche as both neurogenic and non-neurogenic astrocytes are targeted.

## Postnatal/adult LV niche astrogenesis

While it has long been observed that LV NSCs cultured in a dish can differentiate into GFAP^+^ astrocytes, in contrast to neurogenesis, LV niche astrogenesis *in vivo* had been largely ignored. If there is significant baseline astrogenesis from LV NSCs and/or astrogenic progenitors, this will present significant challenges to NSC identification using glial markers since newly generated astrocytes may be indistinguishable. Nestin-CreER lineage-tracing experiments have recently revealed significant astrogenesis from the postnatal LV niche following cortical stroke (Benner et al., 2013; Faiz et al., 2015). While these migrating cells from the niche to cortical regions retain some cellular plasticity (Faiz et al., 2015), they mainly become reactive astrocytes important for normal glial scar formation at the injury site (Benner et al., 2013; Faiz et al., 2015). Additionally, the LV niche can also generate mature astrocytes under physiological conditions (Sohn et al., 2015). Further experimentation would benefit from a set of cellular markers for newborn LV niche astrocytes that are distinct from those used to identify NSCs.

## Neurotransmitter and activity-dependent control in the LV niche

While the actual neural circuitry inputs to the LV niche are poorly understood and an important area for future study, there is mounting evidence that LV niche NSCs are controlled by neurotransmitters and neuronal activity. Several studies have shown that applications of synaptic and modulatory neurotransmitters to the LV niche alter the quantity of proliferative cells. (Banasr et al., 2004; Cooper-Kuhn et al., 2004; Van Kampen et al., 2004; Brazel et al., 2005; Liu et al., 2005; Mudò et al., 2007; O'Keeffe et al., 2009; Alfonso et al., 2012; Paez-Gonzalez et al., 2014; Tong et al., 2014). These results suggest that either the presence of neurotransmitters in the niche causes the release of factors that stimulate NSC proliferation, or that NSCs respond directly to network activity through membrane receptors. Slice electrophysiology experiments using GFAP-GFP reporter mice showed that GFAP^+^ LV astrocytes respond directly to GABA (Liu et al., 2005). Another study performing *in vitro* whole-cell recording chose NSCs based on GFAP-GFP expression combined with the presence of a long cellular projection, and found that local application of serotonin (5HT) caused inward currents in B1 cells that were blocked by 5HT antagonists (Tong et al., 2014). These example studies and others verified the existence of neurotransmitter receptors on GFAP^+^ cells in the LV niche. However, they do not rule out the possibility that non-neurogenic niche astrocytes express the same receptors. Furthermore, GFAP^+^ LV cells have similar resting membrane potentials and input resistances to stellate astrocytes, thus NSCs may not be identified solely based on intrinsic membrane properties (Liu et al., 2005; Lacar et al., 2010; Tong et al., 2014).

We have recently identified a distinct population of cholinergic neurons residing within the postnatal/adult LV niche. Functional experiments utilizing optogenetics to examine circuit connectivity of cholinergic neurons to LV NSCs uncovered neuronal activity-dependent responses in NSCs. NSCs were chosen by a combination of Nestin-CreER lineage-tracing, cellular morphology, and Nestin expression. Acetylcholine (ACh) responses were seen in patch-clamped NSCs following light activation of channelrhodopsin-expressing ChAT^+^ neurons (Paez-Gonzalez et al., 2014). Similar optogenetic activation of ChAT^+^ neurons resulted in no noticeable responses in ependymal niche cells or transiently amplifying Mash1^+^ cells, although there was a consistent response in DCX^+^ neuroblasts. To our knowledge, this may perhaps be the first report of recorded response in a LV NSC as a result of direct neuronal activation. It remains unclear whether differentiated astrocytes in the LV niche have similar capacities to respond to ChAT^+^ neuron activity.

## Can activity response be utilized to define NSCs?

Whether the proliferation and differentiation of LV NSCs can be directly regulated by neural activity is a source of debate. In one view, NSCs are programmed to undergo mitosis and sustain cell division as a part of their identity, and the controlled environment of the niche is protected from outside signals by astrocytic boundaries (Ma et al., 2009). In the olfactory bulb (OB), the main target location for interneurons produced from the LV niche, enhanced sensory activation does not appear to stimulate LV NSC proliferation, suggesting that OB circuit activity is removed from NSC control (Rochefort et al., 2002). In fact, LV proliferation persists following complete bulbectomy (Kirschenbaum et al., 1999). On the other hand, increased LV NSC proliferation is observed after OB neuron cell death (Mandairon et al., 2003), as well as during odor-dependent behaviors such as paternal recognition (Mak and Weiss, 2010) and pheromone mating response (Mak et al., 2007). LV NSCs can also migrate to other brain regions and differentiate into varied cell types in response to cortical injury (Benner et al., 2013; Faiz et al., 2015), demyelination (El Waly et al., 2014), chemical lesions (Aponso et al., 2008), and electrical stimulation (Jahanshahi et al., 2013). Finally, if postnatal/adult NSCs are instructed to produce new neurons or glia for distinct neural circuits, theoretically it would be beneficial for NSCs to be in direct communication with those respective circuits. The finding that local cholinergic neurons can directly innervate LV NSCs is a step toward showing the existence of that neural circuit feedback. It remains possible that these responses may change depending on NSC states in quiescence vs. activation, and future exploration of these neuronal activity-dependent NSC responses may yet provide further refinements to our definitions for postnatal/adult NSC identity.

## Author contributions

All authors listed, have made substantial, direct and intellectual contribution to the work, and approved it for publication.

### Conflict of interest statement

The authors declare that the research was conducted in the absence of any commercial or financial relationships that could be construed as a potential conflict of interest.
